# Faster intervals, faster recoveries - intensified short VO_2max_ running intervals are inferior to traditional long intervals in terms of time spent above 90% VO_2max_

**DOI:** 10.3389/fspor.2024.1507957

**Published:** 2025-01-06

**Authors:** Daniel Fleckenstein, Hannes Braunstein, Nico Walter

**Affiliations:** Department of Endurance Sports, Institute for Applied Training Science, Leipzig, Germany

**Keywords:** HIT, high intensity interval training, running, maximal oxygen consumption, blood lactate, rating of perceived exertion

## Abstract

**Introduction:**

High intensity interval training for improving maximal oxygen consumption (VO_2max_) is a fundamental component of specific preparation phases for middle- and long-distance runners. In this context, short intervals are very popular in practice. The aim of the present study was to determine whether increasing the intensity of short intervals around maximal aerobic speed (vVO_2max_), compared to traditional long interval runs, leads to a greater time spent above 90% VO_2max_.

**Methods:**

12 highly trained middle distance runners (7 males, 5 females) completed two VO_2max_ sessions (4 × 3 min at 95% vVO_2max_, recovery: 3 min at 50% vVO_2max_ vs. 24 × 30 s at 100% vVO_2max_, recovery: 30 s at 55% vVO_2max_) on the treadmill in randomized order. Spiroergometric data, lactate accumulation, heart rate (HR) and perceived exertion was determined. This allowed the recording of time above 90% VO_2max_ and time above 90% HR_max_. To analyze differences between the interval sessions, the paired *t*-test respectively the Wilcoxon test, if data were not normally distributed, were applied.

**Results:**

The time spent above 90% VO_2max_ was significantly lower in the 30-s intervals, despite the higher intensity, compared to the 3-min session (201.3 ± 268.4 s vs. 327.9 ± 146.8 s, *p* = 0.05, *r* = 0.57). In contrast, the time spent above 90% HR_max_ was significantly higher for the 30-s intervals than for the 3-min intervals (820 ± 249 s vs. 545 ± 131 s, *p* < 0.001, *d* = 1.73). The blood lactate concentrations showed higher values in the 3-min session (9.69 ± 1.82 mmol/L) compared to the 30-s session (7.59 ± 2.01 mmol/L, *p* < 0.001, d = 2.34). There was no statistical difference in the rating of perceived exertion between the two sessions (30-s session: 6.5 ± 1.0 vs. 3-min session: 6.8 ± 1.2; *p* = 0.26).

**Discussion:**

The present study showed that intensified 30-s intervals were inferior to traditional 3-min intervals regarding the time spent above 90% VO_2max_. Given the observation of an opposing trend in the time spent above 90% HR_max_, this parameter should be interpreted with caution in traditional training settings.

## 1 Introduction

Most of the training for elite middle- and long-distance runners is performed at low intensity (1–3). However, especially in the Olympic middle distance running events, a part of the training is performed at moderate to high intensity (4). In addition to conventional threshold training (5), interval training sessions aimed at increasing maximal oxygen uptake (VO_2max_) are still completed to enhance performance. The optimal effectiveness of these training sessions are described by research groups as involving a high time spent near maximal oxygen uptake (>90%–95% of VO_2max_) (6, 7), which is also reflected in some international training models (8, 9). Although the optimal intensity range for interval sessions aimed at developing VO_2max_ has not been conclusively scientifically determined (7, 10–12), and overall training volume and altitude training should also be considered, the use of this intensity range in research and practice indicates high relevance.

Several studies (13–15), as well as reviews by Buchheit and Laursen – across disciplines and performance levels (6–16) and Parmar et al. (12), suggest that in running interval training, the use of short interval durations (<60 s) tends to be less successful in achieving high levels of maximal oxygen uptake and maintaining these levels above 90% or 95% of VO_2max_ for comparable durations to longer intervals. For example, Cipryan et al. (13) compared the physiological strain of long intervals (4 × 3 min) vs. short intervals (21 × 30 s) at the same intensity (100% velocity at VO_2max_; vVO_2max_), same recovery intensity (passive), and work-rest ratio (1:1), with the same total duration (21 min) for sixteen highly-trained males, participating in endurance (*n* = 8; VO_2max_: 66.2 ± 5.0 ml/min/kg) or sprint (*n* = 8; VO_2max_: 56.8 ± 5.0 ml/min/kg) events. Averaged over the entire training session, there was an equal relative heart rate, but higher relative oxygen uptake, higher respiratory quotient, and higher subjective exertion in the longer intervals.

In the literature, there are also approaches to optimizing short interval sessions in terms of time spent above 90% VO_2max_. Cipryan et al. (13) recommend increasing the intensity within the intervals, increasing the intensity during the recoveries, or reducing the recovery duration to increase time spent near VO_2max_ when using short interval durations. Millet et al. (17) confirmed in a study with seven highly trained triathletes (VO_2max_: 71.2 ± 4.2 ml/min/kg) the hypothesis that increasing intensity in short interval sessions increases the time spent above 90% VO_2max_. For example, running an interval session with 105% vVO_2max_ (30 s work duration; 30 s recovery; 50% vVO_2max_ recovery intensity), the time above 90% VO_2max_ is about twice as high as running the intervals at 100% vVO_2max_ with the same interval duration, recovery duration, recovery intensity, and total training time (46 ± 20% vs. 23 ± 18% of total training time, or 338.1 ± 149.3 s vs. 167.7 ± 131.3 s; three sets of *n* intervals, with *n* × 30 s = t*_lim_*; 731 ± 121 s total running time).

However, Thevenet et al. showed with nine endurance-trained male adolescents (VO_2max_: 64.9 ± 4.2 ml/min/kg) that under the condition of performing training sessions to maximal perceived exertion, increasing intensity in 30 s intervals (100% maximal aerobic velocity vs. 110% maximal aerobic velocity; 50% vVO_2max_ recovery intensity) does not lead to a significant increase in time spent above 90% VO_2max_ (300.0 ± 150.3 s vs. 197.8 ± 148.1 s) or 95% VO_2max_ (117.8 ± 66.2 s vs. 62.2 ± 74.6 s) (14). Due to the higher anaerobic energy metabolism, the higher intensity results in fewer repetitions (11 vs. 24; 653 ± 187 vs. 1,440 ± 169 s) and therefore a 65% lower time above 90% VO_2max_. The comparison with Cipryan et al. (13) illustrates that the active recovery design by Thevenet et al. (50% vVO_2max_) and the expectedly slightly higher intensity lead to a significant increase in time spent near VO_2max_ in short interval sessions with 100% of the maximal aerobic velocity (14). An increase in time spent near VO_2max_ at higher recovery intensities in short interval sessions, across disciplines and performance levels, was also noted in the review by Buchheit and Laursen (6).

Based on these results, the present study investigated whether a short interval session with higher interval intensity and higher recovery intensity can achieve the same time spent above 90% VO_2max_ as a long interval session with lower intensity during both intervals and recovery periods. Recent studies (13) suggest that increasing intensity in interval and recovery during short intervals could be a method to increase time above 90% VO_2max_. To date, however, there have been no studies in running that have intensified both factors (interval and recovery intensity). The present study addressed this novelty, hypothesising that by intensifying interval and recovery intensity, time above 90% VO_2max_ during shorts intervals can be achieved at an equally high or even higher level compared to long intervals.

## 2 Materials and methods

### 2.1 Participants

12 highly trained middle distance runners (7 males: 24.3 ± 3.4 years, VO_2max_: 62.5 ± 2.1 ml/min/kg, fixed lactate threshold at 3 mmol/L: 4.32 ± 0.26 m/s; 5 females; 19.5 ± 2.3 years, VO_2max_: 54.2 ± 3.0 ml/min/kg, fixed lactate threshold at 3 mmol/L: 4.06 ± 0.43 m/s) took part in the study (18). In the 4 weeks preceding the study, the self-reported training volume was 14.42 ± 3.11 h per week. All runners were part of national training groups and competed in 400 m and 800 m at national and in some cases international championships. The athletes’ personal best times within 6 months before or after the study resulted in a World Athletics Score of 1,058 ± 47 points. They took part in professional running training for 7.0 ± 4.0 years.

The study was conducted within the first 4 weeks following the start of the preseason training phase. At the time of the study, all athletes were free of infections, orthopedic issues or other pain. Athletes were motivated to eat a balanced, carbohydrate-rich diet and encouraged to keep their diet as similar as possible before the tests. The participants were asked to refrain from high-intensity and high-volume training sessions 48 h before the start of the study and were informed in detail about the study design. Moreover, the study is in accordance with the Declaration of Helsinki and was approved by the Ethics Committee of the Institute for Applied Training Science (approval number: ER_2022.19.09_23).

### 2.2 Protocol and test design

Within a period of 5 days, the subjects completed a performance diagnostic and two VO_2max_ interval sessions. On day 1, a VO_2max_ test (ramp test, individual starting speed at lactate threshold, aiming for a total duration of 8–12 min, stage duration: 1 min, increase: 0.15 m/s) was performed to determine maximal oxygen uptake and the speed at which maximal oxygen uptake is reached. In a cross-over design, the runners completed two VO_2max_ interval sessions on the treadmill in a randomized order. An interval session with long interval durations (4 × 3 min at 95% vVO_2max_, recovery: 3 min at 50% vVO_2max_) was compared with a session with short interval durations (24 × 30 s at 100% vVO_2max_, recovery: 30 s at 55% vVO_2max_). A time interval of 48 h was maintained between all tests. During the days between the tests, the athletes were asked to complete only easy training (up to 30 min) or to take a day off. This was documented and care was taken to ensure that all intermediate days were completed individually in the same way. To avoid circadian or shoe-related effects, testing was conducted at the same time of day (±30 min) and with the same running shoes (19). Each participant performed the warm-up before the VO_2max_ test and both training sessions in the same individualized manner. All tests were performed on custom-built treadmills (POMA Maschinen- und Anlagenbau GmbH Poschendorf, Dürrröhrsdorf-Dittersbach, Germany) with 0% inclination. Before the tests, body weight and height were recorded (Seca Vogel and Halke Hamburg 910, seca GmbH and Co. KG, Hamburg, Germany). Respiratory gases were measured spiroergometrically breath-by-breath using a stationary Metalyzer 3B system (CORTEX Biophysik GmbH, Leipzig, Germany). Before the tests and immediately after the end of the test, as well as in the 3rd, 6th and 10th min after the completion of the test, a 20 μl sample of capillary blood was taken from the earlobe, dissolved in 1,000 μl of hemolysis solution, and analyzed to determine lactate accumulation (SUPER GL ambulance system; Dr. Müller Gerätebau GmbH, Freital, Germany). In addition, in the 3-min interval session, a lactate sample was taken after the end of each interval and before the start of the next interval (30 s passive recovery). In the 30-s interval session, samples were taken after the 1st, 2nd, 11th, 12th, 13th, 22nd and 23rd intervals - in order to be able to assess the accumulation of lactate over the entire training session. Heart rate (HR) was continuously measured using a chest strap (Wearlink W.I.N.D.; Polar, Kempele, Finland). Perceived exertion (RPE) was determined at the 3rd, 9th, 15th, 21st min and immediately after the end of each interval session by the modified Borg CR10 scale (20).

### 2.3 Data analysis

All spiroergometric data were processed with the MetaSoft® Studio software (CORTEX Biophysik GmbH, Leipzig, Germany). VO_2max_ and the highest oxygen uptake within the training sessions (VO_2peak_) were defined as the highest value over 15 s (21), further data were averaged over 5 s. The vVO_2max_ was defined, following Billat et al. (22), as the lowest running velocity that elicited VO_2max_ and could be maintained for at least one minute. If an athlete reached VO_2max_ during a stage but was unable to sustain it for 1 min, the velocity from the preceding stage was recorded as their vVO_2max_. This velocity was used as the basis for designing the two following interval sessions. HR data, ventilation (VE) and respiratory exchange ratio (RER) during the VO_2max_ test and interval sessions were averaged over 5 s. The HR data of one subject had to be discarded due to a measurement error. Subsequently, the spiroergometric and HR data were normalized to the maximum value for each subject. This allowed the recording of time above 90% VO_2max_ and time above 90% HR_max_ for each subject individually and for the entire sample. Additionally, the time above 95% VO_2max_ and HR_max_ was calculated. In order to calculate the time above 90% and 95% VO_2max_, the absolute VO_2_ values were used to eliminate the influence of body weight fluctuations over the three test days. The highest blood lactate sample within the training session was recorded as the peak lactate value for data analysis.

### 2.4 Statistical analyses

The descriptive data were given as mean values ± standard deviation (SD). The Shapiro-Wilk test was used to check for normal distribution (*p* > 0.05). To analyze differences in the primary outcomes (time above 90% and 95% VO_2max_, time above 90% and 95% HR_max_, lactate, and perceived exertion) between the interval training sessions, the paired *t*-test respectively the Wilcoxon test (if data were not normally distributed) were applied. For all tests, the significance level was set at *p* < 0.05. Effect sizes of 0.1–0.3 are classified as small, 0.3–0.5 as moderate, and >0.5 as large (23). Data analyses were done using IBM SPSS Statistics 23 (IBM, Armonk, NY, USA). Data visualization was performed using Python Version 3.12 (Python Software Foundation, https://www.python.org/) and the following package Plotly Version 5.22 (Plotly Technologies Inc., https://plotly.com/python/).

## 3 Results

### 3.1 Time spent above 90% and 95% Vo_2max_

**Figure 1:**
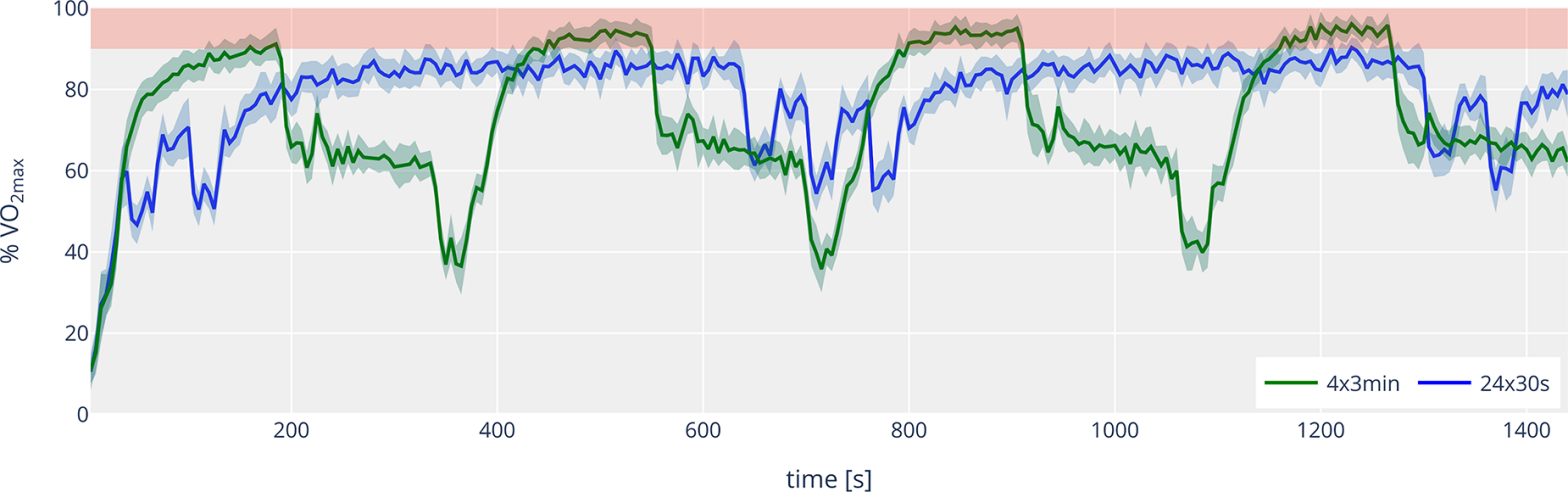
Time course (mean ± 95% confidence interval) of the percentage of oxygen uptake (% VO_2max_) for 4 × 3 min (green line) and 24 × 30 s (blue line). The red area indicates the range between 90 and 100% of VO_2max_.

**Figure 2:**
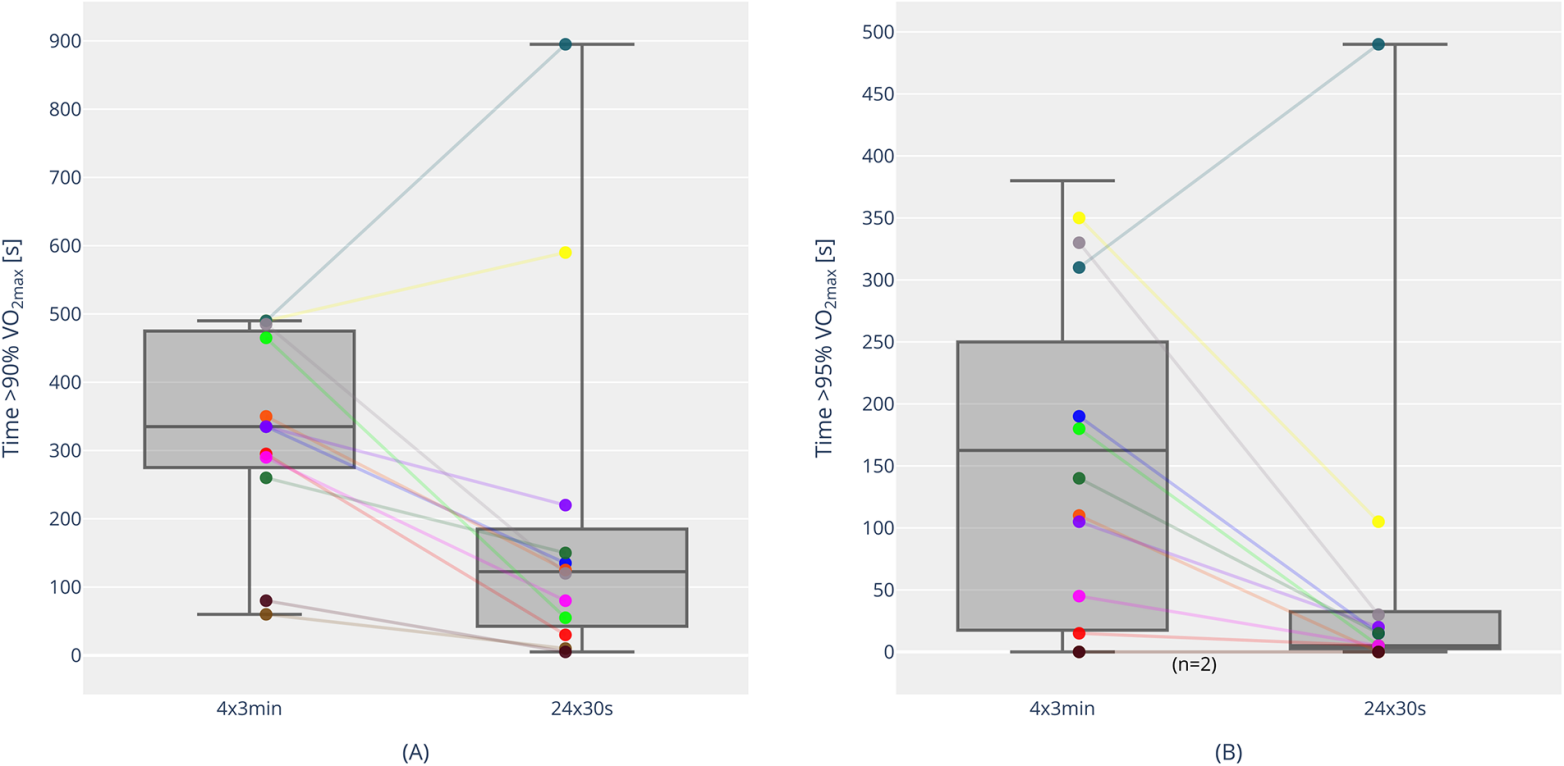
Time above 90% VO_2max_ [**(A)**; individual trends and boxplots with median, interquartile range, individual, minimum and maximum values] and time above 95% VO_2max_ [**(B)** individual trends and boxplots with median, interquartile range, individual, minimum and maximum values] for 4 × 3 min and 24 × 30 s. The color coding corresponds to individual participants in both **(A)** and **(B)** [e.g., Athlete 1 is marked as “yellow” in both **(A)** and **(B)**].

Athletes completed the 30-s session intervals at a running velocity of 5.49 ± 0.34 m/s, with recovery periods at 3.02 ± 0.19 m/s. In the 3-min session, intervals were performed at a speed of 5.21 ± 0.32 m/s, and recovery periods at 2.75 ± 0.17 m/s. The course of the percentage of oxygen uptake (% VO_2max_) across both interval session is shown in Figure 1. The time spent above 90% VO_2max_ was significantly lower in the 30-s intervals, despite the higher intensity, with a high effect size, compared to the 3-min session (201.3 ± 268.4 s vs. 327.9 ± 146.8 s, *p* = 0.05, *r* = 0.57). The time above 95% of VO_2max_ was also lower in the 30-s session than in the 3-min session (57.5 ± 139.2 vs. 147.9 ± 127.4, *p* < 0.05, *r* = 0.57). Individual trends for time above 90% and 95% VO_2max_ are shown in Figure 2. The VO_2peak_ during the training was also significantly lower with 91.2 ± 4.2% of VO_2max_ compared to 97.2 ± 3.9% in the 3-min intervals (*p* < 0.001, *d* = 1.92). However, due to higher oxygen uptakes during the recovery periods and in line with the 5% higher intensity, the short 30-s interval runs had significantly higher mean oxygen uptake over the entire training session (78.1 ± 4.4% vs. 73.0 ± 3.8% VO_2max_, *p* < 0.001, *d* = 2.85).

### 3.2 Time spent above 90% and 95% HR_max_

**Figure 3:**
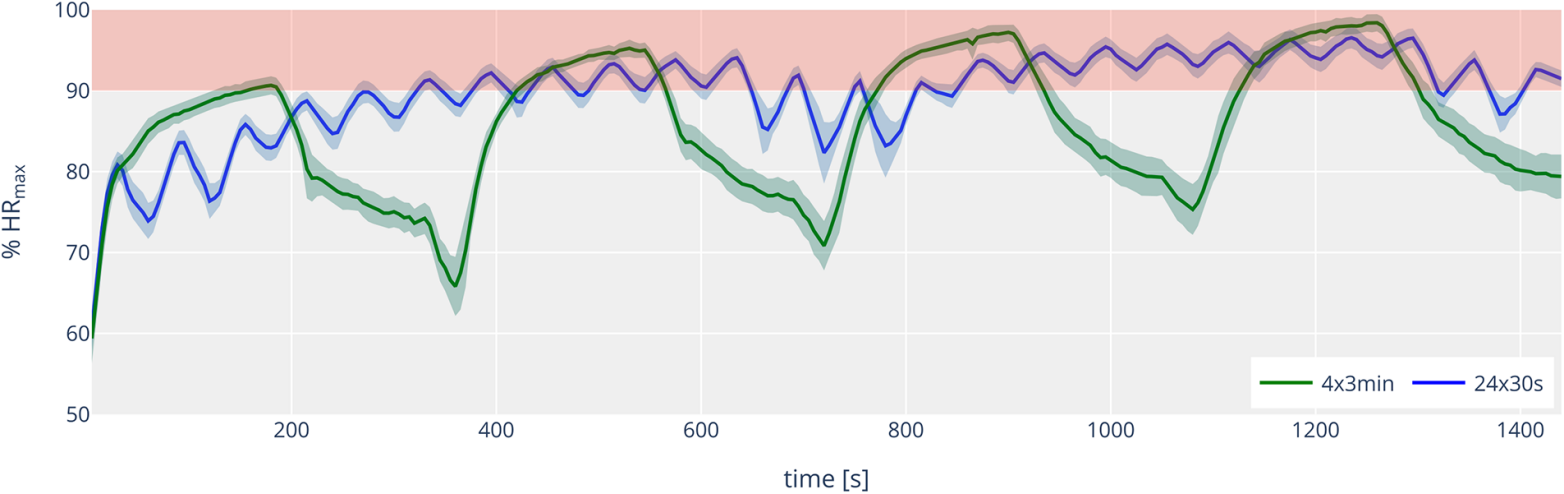
Time course (mean ± 95% confidence interval) of the percentage of heart rate (% HR_max_) for 4 × 3 min (green line) and 24 × 30 s (blue line). The red area indicates the range between 90 and 100% of HR_max_.

**Figure 4:**
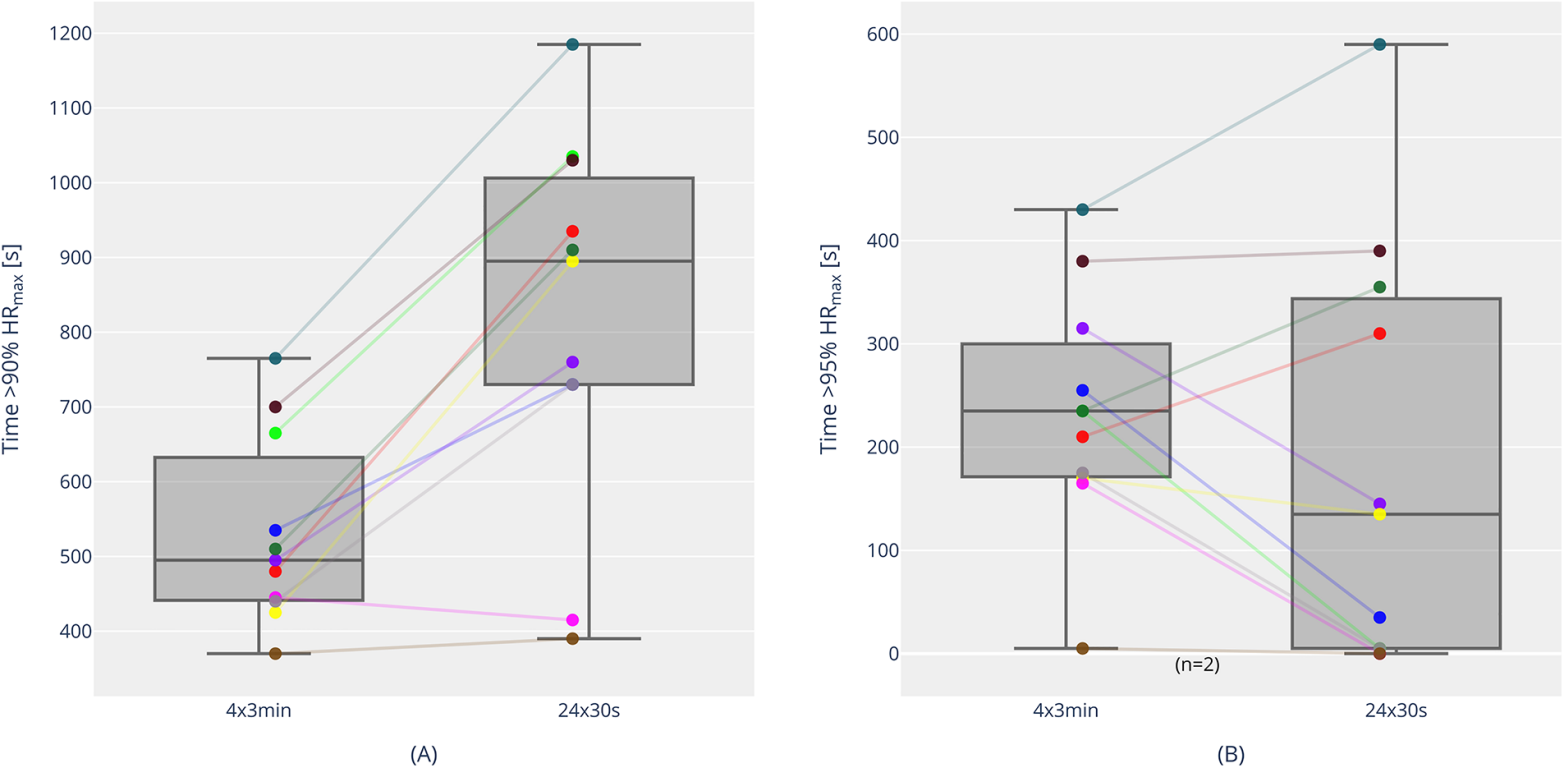
Time above 90% HR_max_ [**(A)** individual trends and boxplots with median, interquartile range, individual, minimum and maximum values] and time above 95% HR_max_ [**(B)** individual trends and boxplots with median, interquartile range, individual, minimum and maximum values] for 4 × 3 min and 24 × 30 s. The color coding corresponds to individual participants in both **(A)** and **(B)** [e.g., Athlete 1 is marked as “yellow” in both **(A)** and **(B)**].

Figure 3 shows the course of the percentage of maximum heart rate (% HR_max_) over the 30-s and 3-min interval sessions. There is also a significant difference between the two sessions, but in a somewhat opposite direction: the time spent above 90% HR_max_ was significantly higher for the 30-s intervals than for the 3-min intervals (820 ± 249 s vs. 530 ± 126 s, *p* < 0.001, *d* = 1.73). The time above 95% HR_max_ showed no statistically significant differences between the 30-s session (179 ± 202 s) and the 3-min session (234 ± 115 s; *p* = 0.155). Individual trends for time above 90% and 95% HR_max_ are shown in Figure 4. Over the entire training session, HR was 89.6 ± 1.9% for the short intervals and 85.5 ± 2.4% for the long intervals (*p* < 0.001, *d* = 3.75). However, the highest 15-s average heart rate (HR_peak_) during the training session was higher in the 3-min session (98.1 ± 1.7%) compared to the 30-s session (96.3 ± 2.1%) (*p* = .001, *d* = 1.51).

### 3.3 Blood lactate concentration

**Figure 5:**
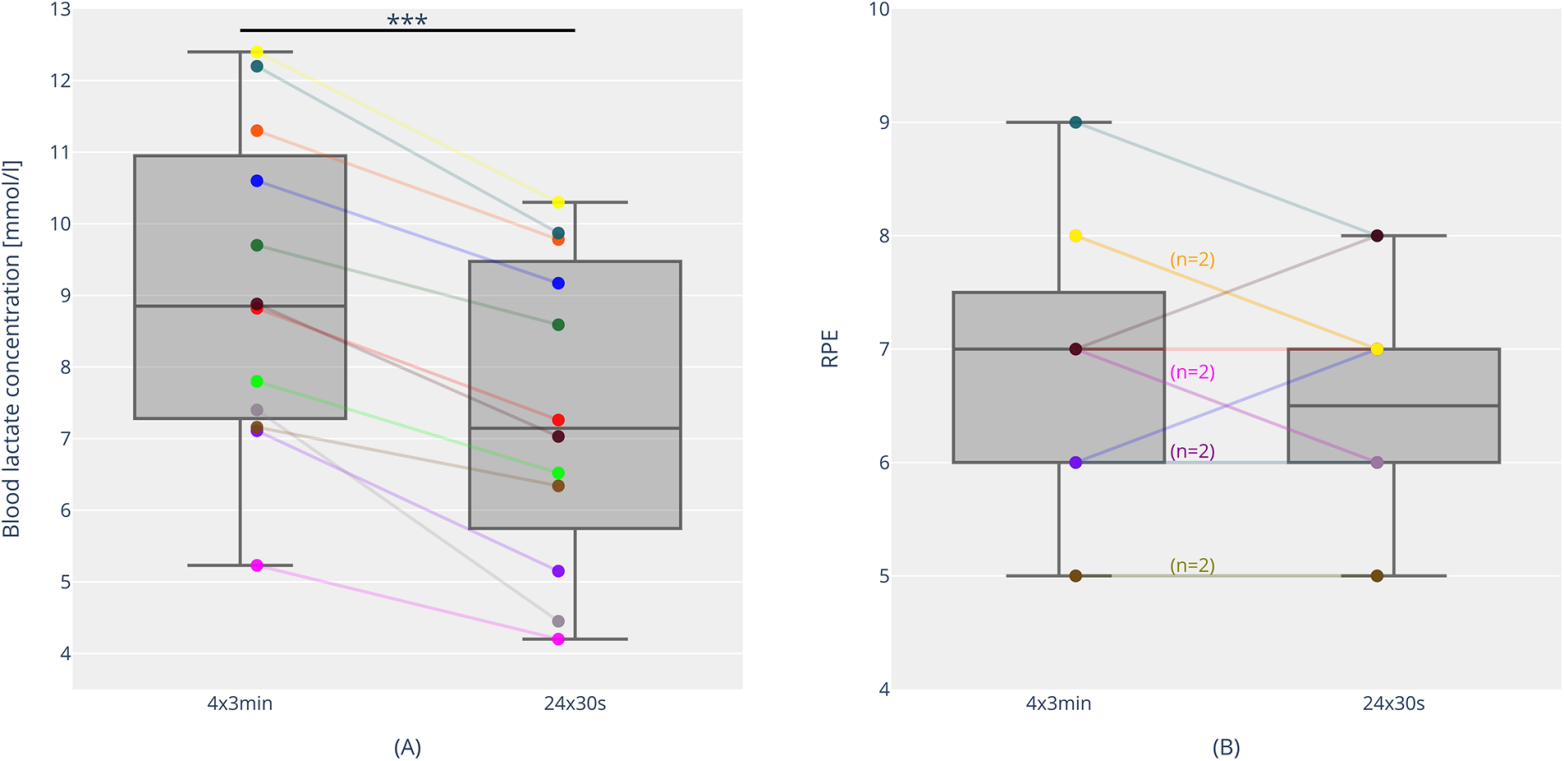
Blood lactate concentrations [**(A)** individual trends and boxplots with median, interquartile range, individual, minimum and maximum values] and rating of perceived exertion [RPE; **(B)** individual trends and boxplots with median, interquartile range, individual, minimum and maximum values] for 4 × 3 min and 24 × 30 s. (*n* = 2): This combination (e.g., “yellow marked”, RPE 8 for 4 × 3 min and RPE 7 for 24 × 30 s) was reported by two participants. *** = *p* < 0.001.

Maximal blood lactate after the VO_2max_ test was 11.19 ± 2.97 mmol/L. The blood lactate concentrations of both interval sessions are shown in Figure 5A. There is a significant difference, with higher maximal lactate values in the 3-min session (9.69 ± 1.82 mmol/L) compared to the 30-s session (7.59 ± 2.01 mmol/L) (*p* < 0.001, *d* = 2.34). There was no noticeable difference in the blood lactate concentrations before the start of the two sessions (*p* = 0.53, *r* = 0.18). During the 30-s session, lactate values were 2.10 ± 0.62 mmol/L (1st interval), 2.94 ± 0.52 mmol/L (2nd interval), 5.61 ± 1.29 mmol/L (12th interval), 5.43 ± 1.38 mmol/L (13th interval), and 7.47 ± 2.03 mmol/L (23rd interval). During the course of the 3-min session, lactate values were 7.02 ± 1.32 mmol/L (1st interval), 8.43 ± 1.00 mmol/L (2nd interval), 8.86 ± 1.38 mmol/L (3rd interval), and 9.42 ± 1.71 mmol/L (4th interval).

### 3.4 Rating of perceived exertion

Figure 5B presents the rating of perceived exertion (RPE) at the end of the two training sessions. There was no statistical difference in the ratings between the two sessions (30-s intervals: 6.5 ± 1.0 vs. 3-min intervals: 6.8 ± 1.2; *p* = 0.26). During the 30-s session, RPE values were 3.1 ± 0.8 (3 min), 4.4 ± 1.0 (9 min), 5.3 ± 1.2 (15 min), and 6.8 ± 1.4 (21 min). During the course of the 3-min session, RPE values were 4.3 ± 1.1 (3 min), 5.4 ± 1.0 (9 min), 6.5 ± 1.4 (15 min), and 6.9 ± 1.3 (21 min).

### 3.5 Ventilation and respiratory exchange ratio

The average ventilation throughout the entire training was 100.1 ± 20.1 L/min in the 30-s session and 98.3 ± 18.6 L/min in the 3-min session, showing no statistical differences (*p* = 0.105). Regarding the 15-s peak ventilation value, the 30-s session showed a lower value (128.2 ± 27.2 L/min) compared to the 3-min session (137.4 ± 29.8 L/min, *p* < 0.05, *d* = 1.14). This difference was also observed in the peak respiratory exchange ratio, which was lower in the 30-s session (1.06 ± 0.05) than in the 3-min session (1.21 ± 0.06, *p* < 0.001, *d* = 2.51).

## 4 Discussion

The main finding of the present study was that intensified 30-s intervals were inferior to traditional 3-min intervals regarding the time spent above 90% VO_2max_. An increase in intensity in the short intervals by 5% in both the interval and the recovery period did not result in a higher effect, despite the same total exercise time and work-rest ratio in both interval forms. These data are consistent with the early results of Astrand et al. in the 1960s (24), who described that long intervals (2–3 min) were the most effective form of interval training and that maximal intensities were not necessary to elicit VO_2max_. Other studies support this preference for long intervals. The findings of Hill and Rowell (25) also align, showing that the minimum time to reach VO_2max_ is at 60% t*_lim_* (running time to exhaustion at maximal aerobic speed). Therefore, some research groups suggest that a duration of 50%–60% *t*_lim_ (∼2–3 min) is ideal for effective VO_2max_ training. In competitive middle- and long-distance running, Cipryan et al. concluded that short intervals are cardiopulmonary less effective than long intervals (13). Both interval forms were performed at 100% vVO_2max_ with passive recovery. A study with eight highly trained triathletes showed that intervals (100% vVO_2max_, recovery: 50% vVO_2max_) of 30 s (recovery: 30 s) led to less time above 90% VO_2max_ than a work duration of 60 s (recovery: 30 s) or ½ *t*_lim_ (recovery: ½ *t*_lim_∼118 s) with the same intensity (26). These results also show, in agreement with our data, a more positive effect with long intervals (>1 min).

The presented results already indicate that there are several factors influencing the interpretation of the “most effective VO_2max_ interval form”. Besides the length of the intervals, the intensity design is also an important parameter (6), both during the intervals and the recovery periods. Our results showed no positive effect of increasing intensity during interval and recovery by 5% in 30-s intervals, however compared to a 3-min session. Millet et al. suggest that a certain increase in interval intensity can be beneficial. The time above 90% VO_2max_ could be increased with 30-s intervals at 105% vVO_2max_ compared to 30-s intervals at 100% vVO_2max_ (with 30-s recovery periods at 50% vVO_2max_) (17). However, a further increase to 110% vVO_2max_ (compared to 100% vVO_2max_) did not lead to a significant difference in time above 90% VO_2max_ under an otherwise identical setting (14), indicating that fine-tuning of speeds seems necessary and faster is not always better.

Additionally, the design of the recovery period (duration, intensity) is another important variable. It determines for example how the VO_2_ drop looks (VO_2_ kinetics) or how lactate metabolism behaves. The 50% vVO_2max_ intensity during recovery in the 3-min intervals led to an average value of 65% VO_2max_ in our study, while in the 30-s intervals, the 55% vVO2max intensity during recovery corresponded to an average of 78% VO_2max_, considering the time lag in VO_2_ increase and decrease. During the 3-min intervals, a significant drop in VO_2_ during the recovery period is evident (Figure 1), which is not the case with the 30-s intervals. On average, the interval and recovery VO_2_ for the short interval runs are almost identical at approximately 78% VO_2max_. Comparing 30-s intervals at 105% vVO_2max_ to exhaustion, Thevenet et al. (21) showed with eight endurance trained adolescents (VO_2max_: 57.4 ± 6.1 ml/min/kg) that active (50% vVO_2max_) and passive recovery periods did not result in a significant difference in time above 90% VO_2max_. However, passive recovery periods required a significantly longer total training time (until *t*_lim_) to achieve this. Dupont et al. (27) demonstrated with eleven male soccer players (VO_2max_: 59.4 ± 4.2 ml/min/kg) with 15 s intervals (15 s at 120% vVO_2max_, recovery: 15 s) that passive recovery periods enabled reaching VO_2max_ and extended *t*_lim_. It should also be pointed out that the “off kinetics” in the recovery periods are not the primary focus of many studies and are therefore not adequately described and recorded in some cases. Currently, the question of the optimal recovery intensity remains unresolved. 50% vVO_2max_ is commonly used, as it is frequently mentioned in the literature and current practice among athletes, and it is assumed to facilitate effective lactate clearance. Studies indicate that the best lactate elimination occurs at intensities around 52%–63% VO_2max_ (28, 29). In the present study, the values for the 3-min session were in this range, while the short recovery periods with increased intensity in the 30-s session were significantly higher, suggesting that “optimal lactate elimination” might not be achieved. However, some of the data collected shows that recovery periods lead to a certain amount of lactate removal. In the 30-s session, the mean value after interval 12 was 5.61 ± 1.29 mmol/L. After interval 13 (i.e., a passive recovery period in between) the value was 5.43 ± 1.38 mmol/L. In the 3-min session, the value dropped from 8.43 ± 1.00 mmol/L after interval 2 to 7.86 ± 1.28 mmol/L during the 3-min recovery period (30 s passive recovery, 2:00 min active recovery).

There are also studies in cycling indicating that short intervals elicit better training adaptations than long intervals. Rønnestad et al. demonstrated this in highly trained cyclists (30). The study by Appelhans in cycling with 12 cyclists and triathletes (VO_2max_: 68.0 ± 6.3 ml/min/kg) shows that the time spent near VO_2max_ can be similar in short interval sessions (3 × 13 × 30 s; recovery 15 s) and long interval sessions (6 × 5 min; recovery: 2:30 min) (31). The results of Rønnestad et al. and Appelhans could highlight the importance of a higher work-rest ratio while using short intervals. Otherwise, these studies raise the question of whether the response to short interval training depends on the specific sport.

In the overall context of VO_2max_ training, a “target time at or near VO_2max_” is often referenced for athletes during a session. This refers to the time spent above 90% VO_2max_ in a single workout. Buchheit and Laursen (6) suggest a minimum of 7 min for team sports athletes and 10 min for long-distance runners or endurance athletes. Based on our results, it becomes clear that most athletes were unable to meet this criterion. On average, the time achieved was about 5.5 min during the 3-min intervals and just over 3 min during the 30-s intervals. However, it is important to note the wide individual range. Some athletes managed to exceed 8 min over 90% VO_2max_ in the 3-min intervals, and one athlete even completed nearly 15 min in the 30-s setting (Figure 2). Moreover, it is important to acknowledge that several other studies have also fallen short of the proposed minimum 7-10 min duration above 90% VO_2max_. Millet et al. (17) achieved approximately 3 min and 6 min, respectively, in their 30-s interval setting (100% vVO_2max_ vs. 105% vVO_2max_). Similarly, Thevenet et al. (14) reported 5 min and just over 3 min (100% vVO_2max_ vs. 110% vVO_2max_). Only one study by Thevenet et al. (21) achieved durations above 90% VO_2max_ of around 12 min and 9 min in their comparison of active and passive recovery during 30-s intervals. However, their athletes ran until exhaustion and completed significantly longer total durations (almost 36 min on average during the passive recovery setting), which is not comparable to our setting (24 min total duration). Overall, it can be concluded that the achieved time above 90% VO_2max_ must also be considered in relation to the total volume of the session. Additionally, the performance level and the associated VO_2_ kinetics of the athletes play a significant role (17). In further analyses, we found no significant correlations between time spent above 90% and 95% VO_2max_ and maximal performance diagnostic parameters (VO_2max_, both relative and absolute; vVO_2max_) or submaximal parameters (fixed lactate threshold of 3 mmol/L; %VO_2max_ at the fixed lactate threshold of 3 mmol/L). Thus, it can be concluded that these parameters cannot be used to infer the effectiveness of a program in terms of time spent above 90% or 95% VO_2max_. It is likely that VO_2_ kinetics and the physiological profile of the athletes (e.g., muscle fiber distribution, different neuromuscular profiles, etc.) play a more significant role (6). One of the highest durations above 90% VO_2max_ was reported in another study by Millet et al. (26). With 60–30 s intervals (100%–50% vVO_2max_), they reached nearly 9 min on average in the “VO_2max_ zone” (26). Nevertheless, in the context of running, the minimum target of 10 min above 90% VO_2max_ per session seems to be an ambitious effectiveness criterion, which has rarely been met in studies and should be reconsidered in future research.

Another interesting finding of the present study was that the time above 90% HR_max_ showed an opposite trend to the time above 90% VO_2max_. The time above 90% HR_max_ was significantly longer in the 30-s session than in the 3-min session (820 ± 249 s vs. 545 ± 131 s), despite the time above 90% VO_2max_ being shorter. This opposing trend was not observed in previous studies by Millet et al. (26). In the comparison of 30–30 s, 60–30 s, and 1/2 *t*_lim_–*t*_lim_ (approximately 118 s), the settings with the longest time above 90% VO_2max_ also recorded the longest times above 90% HR_max_. Additionally, in another study on increased intensity (30–30 s with 100% vs. 105% vVO_2max_), longer durations above 90% VO_2max_ were accompanied by longer durations above 90% HR_max_ (17). It could be speculated that the more intense recovery period may have influenced the development of % HR_max_. Nonetheless, the data indicate that utilization of HR_max_ does not automatically translate to utilization of VO_2max_. This is relevant for the practical work of athletes and coaches, as HR is often used as a key factor in training control. Furthermore, it supports the observations of Laursen and Buchheit (6), who highlight the problems of HR control in high-intensity training. It might also be more appropriate to use the time above 95% HR_max_ as a measure for VO_2max_ load. Interestingly, the time above 95% HR_max_ for the 3-min intervals was, on average, longer than for the 30-s intervals (244 ± 109 s vs. 197 ± 213 s) and might be seen more as the “HR threshold” for high-intensity training in our study. This would align with newer zone models by Haugen et al. (32), which indicate the “HR threshold” for running in classical VO_2max_ training (Zone 5) at 93% HR_max_.

In the present study, differences in blood lactate concentration were observed. With an average peak value of 9.7 mmol/L, the 3-min session was higher than the 30-s session, which had an average value of 7.6 mmol/L. There was also a high degree of individual variability, with maximum values of 12.4 and 10.3 mmol/L in the respective sessions. Internal calculations of the energy system contributions confirmed a slightly higher anaerobic component during the 3-min intervals, despite the lower intensity during the work phase. The lactate values in this study are generally consistent with findings from Cipryan et al. (13). Although no specific values were provided in their study, graphical data indicated peak values of 10–11 mmol/L for 3-min intervals at 100% vVO_2max_, while the 30-s intervals (also at 100% vVO_2max_) showed peak values slightly above 4 mmol/L. It is important to note that Cipryan's study used passive recovery periods, which must be considered when interpreting these results. In contrast, our study used active recovery periods, which is known to stimulate lactate removal and consequently impacts the measured lactate concentrations (33, 34). Other studies investigating 30-s interval sessions report similar lactate values, further corroborating our findings (14, 22). An interesting comparison can be made with the study by Rozenek et al. (35), which examined work-rest intervals of 15–15 s, 30–15 s, and 60–15 s at 100% vVO_2max_ (work) and 50% vVO_2max_ (recovery). In this study, the 15–15 s format produced the lowest lactate values (7.3 ± 2.4 mmol/L), while the 30–15 s (11.5 ± 1.8 mmol/L) and 60–15 s (12.5 ± 1.8 mmol/L) intervals did not differ significantly. This result highlights the impact of the work-rest ratio on peak lactate values, with a ratio of ≥2:1 increasing lactate concentrations even without raising exercise intensity. Moreover the lower lactate values of the 15–15 s format indicates a lower contribution of the anaerobic lactic energy system, what is similar to our study comparing short and long interval runs. Interestingly in short intervals these lower anaerobic lactic energy contribution is not compensated by a higher aerobic energy contribution. Despite 5% higher intensity, the VO_2_ is lower within the short intervals, leading to a lower aerobic energy contribution. Therefore the lack of energy during the short interval runs would inevitably have to be compensated by the anaerobic alactic energy metabolism and its resynthesis during the recovery intervals (36, 37).

In addition to running velocities, HR, and blood lactate concentrations, perceived exertion is often used as a parameter for load monitoring in training settings. Laursen and Buchheit (6) mentioned a threshold of ≥6 on the CR-10 Borg scale for defining high-intensity training, which was also used in the present study. Both interval formats reached this threshold with average values of 6.8 and 6.5, respectively – yet, as previously noted, this did not result in an equally high VO_2max_ stimulus in terms of time spent above 90% VO_2max_. However, it can be concluded that the intensity was not too low, and a subjectively high exertion was achieved. Direct comparisons to other studies are challenging, as most research groups use the 6–20 Borg scale, and converting between the two scales is not straightforward.

### 4.1 Limitations and future research

Although the present study provides valuable insights into the intensification of VO_2max_ intervals, there are several limitations and questions for future research that should be considered. The concept of time spent above 90% VO_2max_ has emerged as a key factor in evaluating the effectiveness of VO_2max_ training. While studies have shown that “traditional VO_2max_ intervals” can lead to VO_2max_ improvements, Stöggl et al. (38) highlight that the effects of HIIT on aerobic parameters remain a subject of ongoing debate and should not be interpreted in a unilateral manner. Furthermore, from a performance perspective, questions remain about how other factors, such as lactate threshold/ventilatory threshold, running economy, or anaerobic capacities, are influenced concurrently. Additionally, neuromuscular adaptations (21) and alactic energy contribution (36, 37) associated with faster and short intervals should also be considered in this context. A holistic approach incorporating these variables should be a key focus of future research and long-term studies. From a methodological standpoint, differences in the determination of vVO_2max_ are also noteworthy. In the present study, an individualized protocol was used for each participant, with VO_2max_ test durations ranging from approximately 6–9 min. In contrast, Millet et al. (17) began their protocol at velocities 8 km/h below the assumed vVO_2max_, leading to calculated test durations of around 15-16 min. This extended time to exhaustion likely resulted in differences in the vVO_2max_ values obtained and suggests that the running velocities used in interval sessions may have been influenced by the testing methodology. Furthermore, the smoothing and averaging of VO_2_ values (5 s, 15 s vs. 30 s) plays a significant role. Going forward, the aim should be to develop comparable protocols for determining “baseline parameters” across studies.

## 5 Conclusion

The results of the study offer several insights that are highly relevant from a practical sports perspective. They highlight that increasing the intensity of short VO_2max_ intervals may not always lead to the desired effect, particularly if achieving a high time spent above 90% VO_2max_ is the primary training goal. Thus traditional long intervals remains superior in terms of time spent above 90% VO_2max_. The organization of intervals (duration, intensity, work-rest ratio, etc.) is crucial in determining the training effects and should be carefully considered by coaches.

The time above 90% HR_max_ shows an opposite trend to the time above 90% VO_2max_ and is higher in short intervals. Moreover the perceived exertion do not differ between short and long intervals despite higher time spent above 90% VO_2max_ in long interval runs. Coaches should be careful not to misinterpret HR and the perceived exertion. In practice, when HR is used as a training monitoring marker, the findings suggest that aiming for HR values exceeding 93%–95% of HR_max_ is essential for effective VO_2max_ training, particularly for maintaining time above 90% VO_2max_. The blood lactate values measured during training should always be interpreted in relation to the design of the specific interval session.

## Data Availability

The raw data supporting the conclusions of this article will be made available by the authors, without undue reservation.
